# Selenized Yeast Protects Against Cadmium-Induced Follicular Atresia in Laying Hens by Reducing Autophagy in Granulosa Cells

**DOI:** 10.3390/cimb46110782

**Published:** 2024-11-18

**Authors:** Caimei Wu, Yuxuan Jiang, Ziyun Zhou, Yuwei Zhang, Yixuan Zhou, Shiping Bai, Jian Li, Fali Wu, Jianping Wang, Yang Lyu

**Affiliations:** 1 Animal Nutrition Institute, Sichuan Agricultural University, Chengdu 611130, China; caimeiwu@sicau.edu.cn (C.W.);; 2 Key Laboratory of Animal Disease-Resistance Nutrition, Sichuan Province, Ministry of Education, Ministry of Agriculture and Rural Affairs, Chengdu 611130, China

**Keywords:** cadmium, selenium, follicular atresia, autophagy, hens

## Abstract

Cadmium (Cd) exposure can induce follicular atresia and laying performance reduction in hens, which is linked to autophagy within the granulosa cells. Selenium (Se) can influence autophagy and counteract Cd toxicity. This study aimed to investigate the protective effect of Se on Cd-induced follicular atresia in laying hens. Sixty-four laying hens were randomly allocated into 4 treatments: control group: basal diet; Se group: basal diet + 0.4 mg/kg Se from selenized yeast; Cd group: basal diet + 25 mg/kg Cd from CdCl_2_; and Cd+Se group: basal diet + 25 mg/kg Cd + 0.4 mg/kg Se. Compared to the Cd group, Se supplementation alleviated the ovarian pathological changes and oxidative stress in the follicles, serum, liver, and ovary, increased daily laying production, ovarian weight and F5–F1 follicle amounts, serum levels of progesterone and oestradiol, and up-regulated mTOR expression (*p* < 0.05), while decreasing the count of autophagic vacuoles, ovarian atresia follicle numbers, and Cd deposition, and down-regulated expression levels of autophagy-related mRNAs, including ATG5, LC3-I, and LC3-II, Beclin1, and Dynein in the follicles (*p* < 0.05). In conclusion, 0.4 mg/kg Se supplementation protected against Cd-induced laying performance reduction and follicular atresia, which were achieved via decreasing oxidative stress and inhibiting mTOR pathways of autophagy.

## 1. Introduction

Cadmium (Cd), a heavy metal, is a ubiquitous environmental contaminant [1]. Its presence stems from a variety of sources, including industrial emissions, agricultural runoff, and the use of cadmium-containing fertilisers [1,2]. These sources release cadmium into the environment, where it can accumulate in soil, water, and air [1,2,3]. As a result, cadmium poses a significant threat to both human and animal health [4]. The detrimental effects of Cd are far-reaching. It can accumulate in living organisms, including humans, leading to various health problems [2,4]. Exposure to cadmium can cause damage to the kidneys, lungs, and bones and has been linked to an increased risk of cancer [1,2,3,4]. It can also negatively impact the reproductive system and immune function [4,5]. The widespread presence of cadmium in our environment underscores the importance of reducing its release and finding ways to mitigate its potential health risks.

The vulnerability of poultry, especially hens, to Cd accumulation is a significant concern. Their feeding habits, often involving the consumption of contaminated feed and water, contribute to increased cadmium intake [6]. This, coupled with their specific physiological processes, facilitates the absorption and retention of Cd within their bodies [6]. Beyond the digestive and circulatory systems, the reproductive organs of female poultry are particularly susceptible to the toxic effects of Cd [7]. This accumulation in reproductive tissues can lead to impaired reproductive function, impacting egg production, fertility, and overall reproductive health [7]. The consequences extend beyond the individual hen, potentially affecting the viability of the entire flock and the poultry industry. Understanding this vulnerability is crucial for developing strategies to mitigate Cd exposure and protect poultry health [6,7]. Growing evidence indicates that Cd accumulation can detrimentally affect ovarian function in female poultry, inducing oxidative stress and subsequent pathological changes and autophagy within the ovary [8,9,10,11]. These effects have been linked to a reduction in egg production. For instance, Cd accumulation has been shown to cause follicular dysplasia, inhibit follicular maturation, and increase follicular atresia in female poultry [9]. Furthermore, a high dietary level of Cd (150 mg/kg) has been demonstrated to induce autophagy in the ovaries of laying hens [10]. In general, these adverse effects underscore the need for effective protection against Cd accumulation in hens and their feed to ensure the safety of poultry and animal products [12].

Selenium (Se), an essential micronutrient, plays a crucial role in various biological processes, including antioxidant defence and immune function [13]. Notably, selenium exhibits a remarkable antagonistic effect against cadmium toxicity [14,15,16,17,18,19,20]. Selenium can reduce cadmium absorption in the digestive tract, preventing its entry into the bloodstream [13,14]. Furthermore, selenium can enhance the detoxification and elimination of cadmium from the body [16,17]. It achieves this by promoting the production of enzymes that bind to cadmium, effectively preventing it from reaching sensitive organs and tissues [13,20]. Se has been shown to mitigate Cd-induced injury and oxidative stress in the ovaries of female poultry [14], reducing Cd deposition in ovarian tissue [15]. Furthermore, Se attenuates Cd-induced damage in vital organs such as the liver, kidney, and testis, which are particularly susceptible to Cd-induced oxidative stress [16,17]. Supplementation with Se has been associated with improved productive performance in female poultry [18]. It is important to note that Se can also influence autophagy. Se deficiency has been linked to enhanced autophagy, potentially leading to liver damage [19,20,21]. For instance, Frustaci et al. (2012) observed that Se deficiency can increase cardiomyocyte autophagy [19]. Conversely, Se supplementation has been shown to attenuate Cd-induced autophagy in the ovarian tissues of laying hens by inhibiting autophagy and modulating energy metabolism [9,20]. These results imply the potential benefits of Se supplementation in mitigating the deleterious effects of Cd toxicity in poultry.

Nevertheless, the current research on the role of Se supplementation in protecting against Cd toxicity in laying hens is limited. The underlying mechanisms by which Se alleviates Cd toxicity on ovarian function and autophagy remain to be fully elucidated [20]. To address these knowledge gaps, this study was undertaken to investigate the protective effects of Se supplementation by using selenized yeast on Cd-induced follicular atresia in laying hens. Moreover, the study aimed to unravel the pivotal role of autophagy in follicular granulosa cells in the context of Cd toxicity and Se supplementation. The findings of this study are anticipated to provide a theoretical basis for understanding the role of autophagy during Cd accumulation in laying hens, while also offering practical implications for the use of Se supplementation in mitigating Cd toxicity in poultry.

## 2. Materials and Methods

### 2.1. Materials

Cadmium chloride (CdCl_2_), analytically pure, was purchased from the Kelong company (Chengdu, Sichuan, China). Selenized yeast, which contains about 60% Se-Met, was provided by Chelota biotechnology Co., Ltd. (Deyang, China). Laying hens were obtained from commercial layer farms (Mianyang, China). The test site was the farm of Sichuan Agricultural University (Ya’an, China). All experimental procedures were approved by the Animal Care and Use Committee of Sichuan Agricultural University (Chengdu, China, approval code: 20220229).

### 2.2. Experimental Design

A total of 64 healthy Lohmann pink-shell laying hens (1.42 ± 0.12 kg body weight, 63 weeks of age) were evenly divided into four groups based on a similar egg-laying frequency (i.e., 64.37%), with two replicates of eight hens each. A cage served as one replicate unit, housing two hens per cage. A corn-soybean meal basal diet was formulated according to the National Research Council guidelines [21], as previously described [22]. The treatments were as follows: the control group (CON): hens fed the basal diet; the Se group (Se): basal diet supplemented with 0.4 mg/kg Se, derived from selenized yeast; the Cd group (Cd): basal diet supplemented with 25 mg/kg Cd, derived from CdCl_2_; and the Cd+Se group (Cd+Se): basal diet supplemented with 25 mg/kg Cd and 0.4 mg/kg Se. The Se dosage was selected based on the Chinese regulatory limit of 0.5 mg/kg Se, and the Cd dosage was chosen according to our previous study as well as the existing literature reports on adaptive toxicity doses [9,20,22,23].

The trial duration was 8 weeks. All hens were housed in a room maintained at a controlled ambient temperature of 22 ± 2.0 °C and relative humidity of 65 ± 2.0%. Feed and water were provided ad libitum, with a photoperiod of 16 h light: 8 h dark. Hen-day production was recorded every day.

On day 56 of the trial, blood and serum samples were collected according to our established standard [22]. All hens were sacrificed by cervical dislocation; ovaries were harvested immediately after washing with phosphate-buffered saline (pH = 7.2–7.4). Then, follicles, ovarian tissue, and layers of follicles were collected from each hen and stored at −80 °C until analysis. In order to analyse ovarian follicle counting and histopathology, a portion of the ovarian tissues were placed in 4% paraformaldehyde (pH = 7.2) fixation and paraffin; small white follicle (SWF, 2–4 mm), large white follicle (LWF, 4–6 mm), and small yellow follicle (SYF, 6–12 mm) were obtained, finally. Frozen follicles were ground to powder, mixed with medium at a ratio of 1:9, and centrifuged at 1200× *g* for 15 min; the supernatant was then collected for further analysis.

### 2.3. Analytical Methods

Serum and follicular concentrations of oestradiol (E2), progesterone (P4), and reactive oxygen species (ROS) were determined by ELISA using a commercially available chicken-specific kit (Enzyme- linked Biotechnology Co., Ltd., Shanghai, China), following the manufacturer’s instructions.

Antioxidant capacity of hens was analysed by spectrophotometry-based commercial kits (Jiancheng Bioengineering Institute, Nanjing, China) according to protocol instructions, including the levels of malondialdehyde (MDA) and glutathione (GSH) and the activities of total superoxide dismutase (T-SOD), glutathione s-transferase (GST), and glutathione peroxide (GPx) in the serum, follicles, ovaries, and liver.

Follicular pathological sections were prepared by H&S stain, and follicle counting was carried out through microscopic inspection using our established standard [23,24]. Briefly, primordial follicles were defined as those with oocytes surrounded by a flattened layer of 5 to 8 somatic cells. Antral follicles, on the other hand, contain more than one layer of granulosa cells and are further categorised into SWFs (2–4 mm), LWFs (4–6 mm), and SYFs (6–8 mm). Antral follicles are characterised by an oocyte surrounded by a layer of antral granulosa cells containing an expanded cell or a whole layer of cuboid cells.

Cadmium concentrations in the ovary and follicles were determined using a ContrAA 700 high-resolution continuous light source atomic absorption spectrometer (Analytik Jena AG, Jena, Germany). Selenium concentrations in the ovary and follicles were measured using an AFS-230E dual-channel atomic fluorescence photometer (Haiguang Instrument, Beijing, China). The preparation protocol for Cd and Se determination followed previously established methods [22].

The expression levels of autophagy-related mRNAs were determined using quantitative real-time polymerase chain reaction (qPCR), including mTOR, Beclin1, ATG5, Dynein, LC3-I, and LC3-II. Total RNA was extracted using TRIzol lysis buffer, followed by reverse transcription. The qPCR reaction was performed using TB Green^TM^ Premix Ex Taq^TM^ II (Novozan, Nanjing, China) according to the manufacturer’s instructions. A CFX96 well real-time fluorescence qPCR instrument was used to detect the expression levels of the target genes. The qPCR results were analysed using the 2^−ΔΔCt^ method. A reference gene (GAPDH) was used to normalise the data. The primer sequences for the target genes and the reference gene are listed in Table S1.

The ultra-structure of the granulosa cell layer of follicles was analysed by transmission electron microscopy (TEM, JEM-1400PLUS, JEOL Ltd., Tokyo, Japan). Follicular membranes were gently cut open using a scalpel, and the follicular membrane and granulosa layer were carefully separated using forceps. The granulosa layer was then placed in a TEM tissue-specific fixative (3% glutaraldehyde for pre-fixation and 1% osmium tetroxide for re-fixation). Samples were subsequently dehydrated in a graded acetone series. After dehydration, the samples were sequentially passed through different ratios of dehydrating agent and epoxy resin permeant, with a dehydration time of 30–60 min. The permeated sample blocks were then placed in appropriate moulds and filled with embedding solution. After heating, the samples were sliced using an ultrathin slicer. Ultrathin sections, approximately 50 nm thick, were floated on the liquid surface of the knife bath and then transferred to copper mesh grids. The sections were stained with uranyl-acetate and lead citrate for specific durations and then examined using TEM.

### 2.4. Statistical Analysis

Data analysis was performed using SAS software (version 9.4, SAS Institute Inc., Cary, NC, USA). Hen-day production data were analysed using the PROC MIXED procedure. The model was: *Y*_ijk_ = *µ* + *T_i_* + *P_j_* + (*TP*)*_ij_* + *e_ijk_*; where *Y* was an observation of the dependent variable, *μ* was the population mean for the variable, *T_i_* was the fixed effect of treatment (CON, Se, Cd, or Cd+Se), *P_j_* was the period effect, the *TP* was the interaction between dietary treatment and period, and *e* was the random error associated with the observation. The remaining data were analysed using one-way analysis of variance with the GLM procedure. Multiple comparisons between different treatments were conducted using the Duncan’s method. All data are presented as mean ± standard error of the mean (SEM). Statistical significance was set at *p* < 0.05.

## 3. Results

### 3.1. Ovarian Histology and Follicle Counts

**Figure 1:**
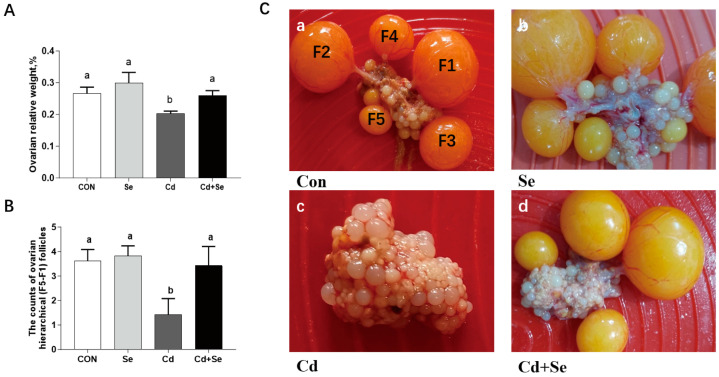
Effects of Cd and Se supplementation on the morphological changes in follicles and ovarian weight in laying hens. (**A**) Ovarian relative weight; (**B**) number of F1–F5 follicles; (**C**) ovarian morphology: a—the control (Con) group, b—the Se group, c—the Cd group, and d—the Cd+Se group. Different letters (a and b) on the bars represent significant differences (*p* < 0.05), data are means ± SEM (*n* = 8).

The 25 mg/kg of Cd supplementation resulted in a significant decrease in ovarian relative weight (Figure 1A) and a reduction in the number of F5-F1 follicles compared to the CON group (*p* < 0.05) (Figure 1B,C). The Se supplementation of 0.4 mg/kg effectively mitigated these Cd-induced effects, demonstrating significant improvements compared to the Cd treatment group (*p* < 0.05).

**Figure 2:**
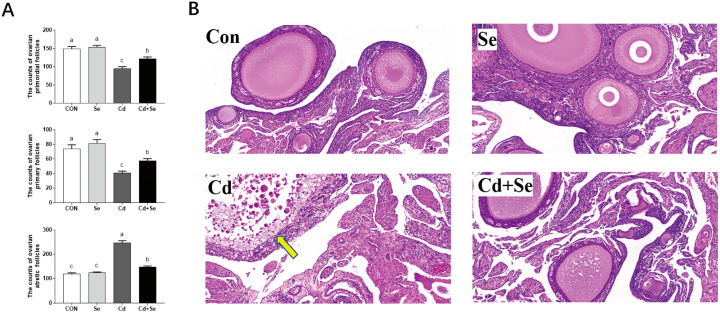
Effects of Cd and Se supplementation on the counts of primordial follicles, primary follicles, and atretic follicles (**A**), as well as on ovarian histomorphology (**B**) in laying hens aged 63 to 70 weeks. (**B**) The histological images were magnified 200 times, and the yellow arrow indicates that the ovarian vacuolar cells are clearly visible. Different letters (a, b, and c) on the bars represent significant differences (*p* < 0.05), data are means ± SEM (*n* = 8).

The Cd supplementation led to a decrease in primordial follicles and primary follicles, while simultaneously increasing the number of atretic follicles (*p* < 0.05) (Figure 2A). This resulted in a decline in egg production performance (Table S2). Cd exposure also exacerbated ovarian morphology, with clear visibility of ovarian vacuolar cells (Figure 2B). However, Se supplementation demonstrated a mitigating effect on these aspects.

### 3.2. Anti-Oxidative Capacity

**Figure 3:**
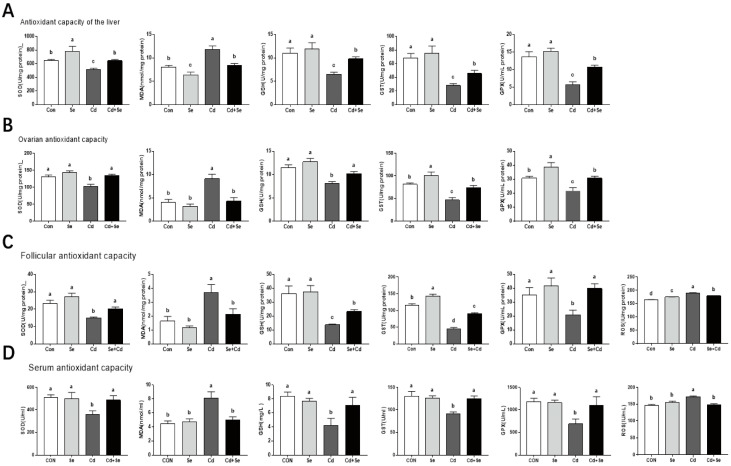
Effect of Cd and Se supplementation on the antioxidant capacity in the liver (**A**), ovarian (**B**), follicular (**C**) and serum (**D**). Different letters (a, b, c, and d) on the bars represent significant differences (*p* < 0.05), data are means ± SEM (*n* = 8).

Figure 3 presents the results of antioxidant capacity analysis in the liver (A), ovary (B), follicles (C), and serum (D). Compared to the CON group, the Cd treatment significantly decreased the levels of GSH and the activities of GPx, GST, and SOD while increasing the levels of MDA in the liver, ovary, follicular, and serum (*p* < 0.05). The Se supplementation showed opposite changes in these parameters, which significantly increased the levels of GSH and the activities of GPx, GST, and SOD while decreasing the levels of MDA in all the four samples (*p* < 0.05).

### 3.3. Serum and Follicular Hormones

**Figure 4:**
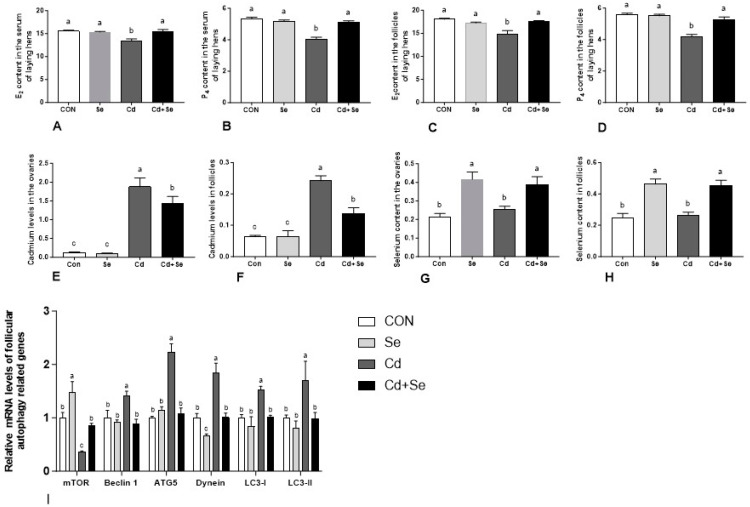
Effects of Cd and Se supplementation on serum and follicular hormones, ovarian and follicular deposition of Cd and Se, and follicular autophagy-related mRNA expression levels. (**A**) E2 levels in the serum; (**B**) P4 levels in the serum; (**C**) E2 levels in the follicles; (**D**) P4 levels in the follicles; (**E**) ovarian Cd deposition; (**F**) follicular Cd deposition; (**G**) ovarian Se deposition; (**H**) follicular Se deposition; (**I**) follicular autophagy-related mRNA expression levels. Different letters (a, b and c) on the bars represent significant differences (*p* < 0.05), data are means ± SEM (*n* = 8).

The results of hormone in the serum and follicles are shown in Figure 4A–D. Compared to the CON group, Cd treatment significantly reduced the levels of E2 and P4 in the serum (Figure 4A,B) and follicles (Figure 4C,D) (*p* < 0.05). The Se supplementation significantly increased the levels of these two hormones in the serum and follicles compared to the Cd group (*p* < 0.05).

### 3.4. Cd and Se Deposition

Figure 4E–H presented data on the effects of Cd and Se supplementation on the deposition of Cd and Se in the ovary and follicles. Compared to the CON group, the Cd treatment significantly increased the deposition of Cd in the ovary (Figure 4E) and follicles (Figure 4F) (*p* < 0.05). The Se supplementation significantly increased the deposition of Se in the ovary (Figure 4G) and follicles (Figure 4H) compared with Cd treatment group (*p* < 0.05).

### 3.5. Expression Levels of Autophagy-Related mRNAs

Results of the relative expression levels of autophagy-related mRNAs were presented in Figure 4I. Compared to the CON group, Cd treatment significantly up-regulated the relative expression levels of Beclin1, ATG5, LC3-I, LC3-II, and Dynein and down-regulated the expression levels of mTOR in the follicles (*p* < 0.05). Compared to the Cd group, Se supplementation significantly down-regulated the expression of Beclin1, ATG5, LC3-I, LC3-II, and Dynein up-regulated the expression levels of mTOR in the follicles (*p* < 0.05).

### 3.6. Ultrastructure of Granulosa Cell Layer of Follicles

**Figure 5:**
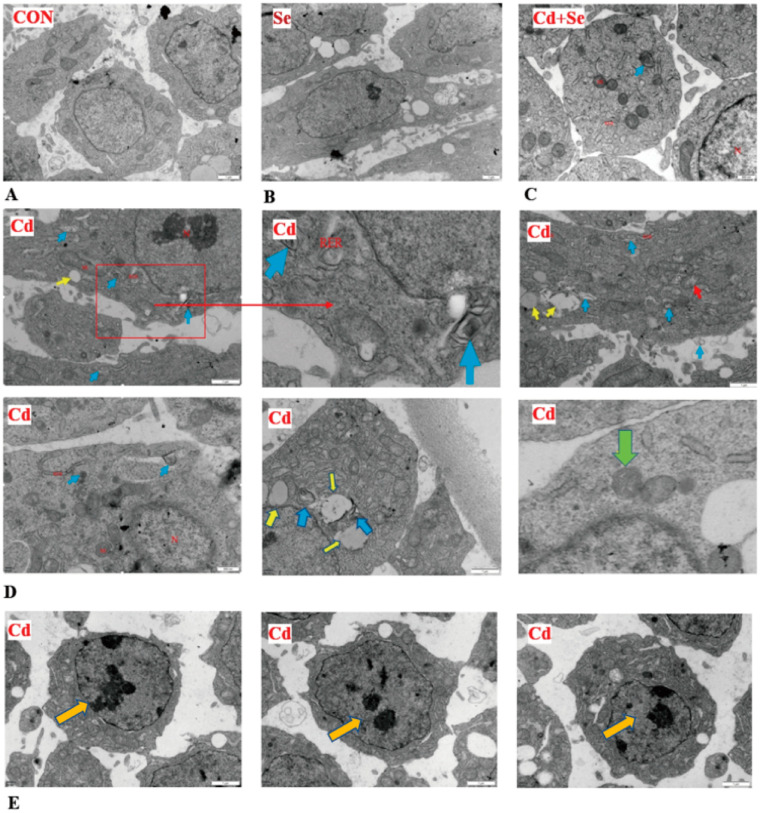
Effects of Cd and Se supplementation on ultrastructure of granulosa cell layer of follicles. (**A**) The CON; (**B**) Se supplementation; (**C**) Cd+Se supplementation; (**D**,**E**) Cd treatment. The red box and lines in the first picture of (**D**) indicate that the next picture is an enlarged part of the boxed area. Blue arrows represent autophagosomes, yellow arrows represent lipid droplets, red arrows represent mitochondria that have swollen, the green arrow represents mitochondrial autophagy, and orange arrows represent apoptosis. N, nucleus, Mi, mitochondria, RER, rough endoplasmic reticulum.

As shown by the TEM analysis (Figure 5), a greater number of autophagic vesicles were observed in the Cd group compared to the CON group, whereas the Se supplementation resulted in a reduction in the count of autophagic vesicles when compared to the Cd group.

## 4. Discussion

The efficacy of egg production is contingent upon the functional integrity of the reproductive system, with the ovaries and follicles being paramount [25]. Specifically, the prevalence of atretic follicles exerts a direct influence on poultry production performance [26]. The primary causative factor for the decline in laying rate induced by Cd is follicular atresia, triggered by dietary Cd exposure [27]. Follicular atresia is characterised by heightened autophagy within the granulosa cells of ovarian follicles, potentially leading to aberrant cellular development and contributing to systemic inflammation and other pathologies [28,29]. Exposure to Cd has been demonstrated to induce autophagy in various tissues, stimulating the expression of mRNA and protein associated with Beclin1, LC3-I, and LC3-II [30,31,32]. Furthermore, Cd exposure triggers the production of ROS within the follicles, activating the mechanistic target of the mTOR autophagy pathway [33,34]. The mTOR pathway typically inhibits the initiation of autophagy, thereby preventing the formation of autophagosomes [35]. Our study corroborates this, revealing elevated ROS levels in both follicular and serum samples following Cd exposure. This oxidative stress within the follicles promoted the expression of the aforementioned autophagy-related mRNAs. Furthermore, TEM analysis revealed the presence of characteristic autophagosomes in Cd-exposed follicles. Collectively, these findings suggest that Cd accumulation contributes to a decline in daily egg production in hens, potentially linked to follicular atresia triggered by autophagy.

Our research indicated that Cd accumulation occurs not only in the ovaries but also within the follicles, albeit at lower concentrations in the latter. This disparity may be attributed primarily to the barrier function of the follicular wall, which impedes the osmotic deposition of Cd [36]. Notably, E2 and P4, crucial reproductive hormones secreted by the ovary, play a vital role in follicle development [37]. Cd is recognised as a classic environmental oestrogen [38] and has been shown to inhibit the release of E2 and P4 from the ovary, directly impairing hormone secretion and negatively impacting ovarian function [38,39]. Our findings align with previous reports demonstrating that Cd reduces P4 and E2 levels within the follicles. This observation suggests that Cd induces endocrine disruptions in the reproductive system, resulting in an increased prevalence of atretic follicles and a decline in egg production performance. While our study was conducted with a limited number of hens, existing literature supports the reliability of our findings [8,40]. In general, oxidative stress appears to be a key mechanism underlying Cd-induced autophagy within follicular granulosa cells, ultimately contributing to follicular atresia and reduced reproductive performance.

In contrast to the detrimental effects of Cd, supplementation with 0.4 mg/kg Se demonstrated a decrease in the number of atretic follicles, ultimately leading to an increase in daily egg production. These findings are consistent with several recent studies [10,13,41,42]. The inhibition of autophagy appears to be a pivotal mechanism by which Se reduces atretic follicles and enhances laying production. Se has been shown to alleviate Cd-induced autophagy in the ovarian tissues of laying hens by suppressing autophagy and modulating energy metabolism [9]. A study by Wenzhong et al. (2017) revealed that Se deficiency resulted in elevated expression of autophagy-related genes [43]. Emerging research has highlighted the mTOR signalling pathway as a key regulator of autophagy [44,45]. Autophagy is a cellular process that dismantles and recycles cellular components [44], providing essential nutrients and energy during periods of stress [46], such as exposure to Cd toxicity [9,43]. Conversely, when nutritional and/or health conditions are favourable, mTOR activity is elevated, suppressing autophagy to facilitate cell growth and proliferation [44,45]. Our findings similarly indicated that Se supplementation decreased both autophagosome formation and overall autophagy levels in follicular tissues. This effect was likely due to a multi-pronged mechanism, including the regulation of the mTOR pathway as well. Firstly, Se activated mTOR and suppressed Beclin1 mRNA expression, which collectively slowed down the initiation and expansion of autophagy. Secondly, Se down-regulated the expression of ATG5, LC3-I, and LC3-II mRNAs, thus hindering the formation of autophagosomes and affecting the processes of nucleation and maturation within the autophagic pathway. Lastly, Se down-regulated Dynein expression, hindering the movement of autophagosomes towards lysosomes, ultimately leading to a reduction in overall autophagy levels.

Furthermore, Se increased the ovary-to-body weight ratio, ameliorated pathological changes within the ovaries, and enhanced the activity of antioxidant enzymes, thereby reducing MDA and ROS levels. Numerous studies have demonstrated that Se can protect tissues from Cd toxicity by reducing Cd accumulation within tissues and mitigating Cd-induced oxidative stress, apoptosis, and autophagy [8,9,10,11,30,47]. This protective effect is likely attributed to the ability of Se-Met to bind Cd and form an insoluble complex, effectively antagonising Cd toxicity [48]. In addition, Se is a vital component of several enzymes, including various antioxidant reductases and selenoproteins [49]. This inherent role grants Se potent antioxidant properties, enabling it to scavenge oxygen radicals and influence redox regulation [47,49]. Furthermore, Se can enhance the efficiency of enzymes critical for cellular processes, such as GPX [14]. Se achieves this by utilising selenocysteine within the GPX active site to eliminate free radicals, including hydrogen peroxide and phospholipid hydrogen peroxide [50].

Moreover, this study further observed that Se can accumulate within the ovaries and follicles of laying hens, effectively antagonising Cd toxicity. Se readily reaches the reproductive system of laying hens, not only increasing the secretion of E2 and P4 from follicles but also mitigating oxidative stress within the ovaries and follicles. This includes reducing ROS and MDA levels as well as decreasing the number of atretic follicles. These effects collectively improve ovarian function, egg production performance, and counteract the reproductive toxicity of Cd in laying hens. Finally, the findings of this study not only underscore the benefits of Se supplementation in mitigating Cd toxicity in poultry but also offer a promising avenue for reducing the adverse effects of Cd exposure in humans. Beyond reducing chronic exposure through safer egg products by improving poultry health, this study presents a potential experimental model for investigating Cd toxicity on the human reproductive system. Future research could focus on preventing and treating Cd toxicity in humans, particularly women, by utilising a laying hen model.

## 5. Conclusions

This study evaluated the protective effects of Se supplementation (dived from selenized yeast) on Cd-induced follicular atresia in laying hens. In summary, Cd exposure at 25 mg/kg (derived from CdCl_2_) significantly decreased laying production and increased follicular atresia in laying hens. These negative effects were correlated with increased oxidative stress (i.e., increased MDA and decreased GSH, GPx, GST, and SOD) and autophagy within the follicles and ovaries (i.e., a greater number of autophagic vesicles). However, supplementation with 0.4 mg/kg of Se effectively protected against the Cd-induced decline in laying production and follicular atresia. This protective effect was associated with enhanced antioxidant capacity and the inhibition of autophagy in the follicular granulosa cells. Further mechanistic analysis indicated that the observed reduction in autophagy were likely mediated by the up-regulation of the mTOR signalling pathways and concomitant down-regulation of autophagy-related mRNAs (i.e., Beclin1, ATG5, LC3-I, LC3-II, and Dynein).

## Data Availability

The data supporting the findings of this study are included within the article. Further inquiries can be directed to the corresponding author.

## Supplementary Materials

The following supporting information can be downloaded at: https://www.mdpi.com/article/10.3390/cimb46110782/s1, Table S1: Gene-special primers for qPCR; Table S2: The effects of Cd and Se supplementation on hen-day production of laying hens.
