# Supplementary material for: Selenized Yeast Protects Against Cadmium-Induced Follicular Atresia in Laying Hens by Reducing Autophagy in Granulosa Cells
Source: Curr Issues Mol Biol. 2024 Nov 18;46(11):13119–30. doi: 10.3390/cimb46110782 (PMC11592890; doi:10.3390/cimb46110782)
Supplement: Supplementary file 1 [file cimb-46-00782-s001.zip › cimb-3301289-supplementary.pdf]

Selenized yeast protects against cadmium-induced follicular atresia in laying hens by reducing autophagy in granulosa cells

Supplementary Materials:

Table S1. Gene-special primers for qPCR.

Gene	Orientation	Primer sequence (5′-3′)	Accession number	Product size
mTOR	Forward	CATGCCAAAAGGTCTTGCCC	XM_040689168.1	240
	Reverse	AAGTGGATGGAAGGCGTGAG		
Beclin1	Forward	AGAACCAGATGCGTTATGCTCA	NM_001006332.1	129
	Reverse	GGAGACGGCCAAGTCTGAAG		
ATG5	Forward	GCTATCACCCCTGAAGATGGAG	NM_001006409.2	76
	Reverse	GTGGTGTTTCCAGCATTGGC		
Dynein	Forward	CCTCGGCCAAGTCGCTATTC	NM_001277879.1	115
	Reverse	AAGAAGGCTGAAGGAACTGGG		
LC3-I	Forward	TTACACCCATATCAGATTCTTG	XM_417327.6	143
	Reverse	ATTCCAACCTGTCCCTCA		
LC3-II	Forward	CCCCACAAAGATCCCGGTAAT	XM_040666873.1	89
	Reverse	CCTCGGGGACGAGAAACTTG		
GAPDH	Forward	GGGGAAAGTCATCCCTGAGC	NM_204305.2	111
	Reverse	TTGGCTGGTTTCTCCAGACG		

Table S2. The effects of Cd and Se supplementation on hen-day production of laying hens.

**Item**	**stage (weeks）**	**Main effect of diet (mg/kg)**				**SEM**	**Main effect of time/weeks**		**SEM**	**P -Value**		
Item	Time/weeks	CON	Se	Cd	Cd+Se	SEM	1～4	5～8	SEM	Treat	Time	Treat* Time
Hen-day production/%	1～4w	86.83a	87.94a	87.15a	87.29a							
	5～8w	86.66a	86.43a	77.28b	85.28a							
	Overall	86.74	87.19	82.21	86.29	0.431	87.30a	83.91b	0.280	<.0001	<.0001	<.0001

Values with different letter superscripts mean significant difference ($P < 0.05$). Data show the mean ± SEM. Other laying performance were not listed in afore table, because the number of hens used in this study was limited.
